# Evaluation of acute-to-chronic ratios of fish and *Daphnia* to predict acceptable no-effect levels

**DOI:** 10.1186/s12302-016-0084-7

**Published:** 2016-05-12

**Authors:** Martin May, Wiebke Drost, Sabine Germer, Tanja Juffernholz, Stefan Hahn

**Affiliations:** 1 Fraunhofer Institute for Toxicology and Experimental Medicine, Chemical Risk Assessment, Nikolai-Fuchs-Strasse 1, 30625 Hannover, Germany; 2 German Federal Environment Agency, Wörlitzer Platz 1, 06844 Dessau-Rosslau, Germany

**Keywords:** Aquatic toxicology, Environmental hazard assessment, Acute-to-chronic extrapolation, REACH

## Abstract

**Background:**

Acute-to-chronic extrapolation is an important approach to predict acceptable no-effect levels from acute data which has some uncertainties, but is valuable for risk assessment of chemical substances. With regard to the still limited and heterogenic data of chronic fish tests, conclusions on aquatic hazard estimation need to be checked and the question arises whether the chronic toxicity to fish can be adequately derived from acute data. A comprehensive dataset including ecotoxicological studies of 203 substances was used to investigate acute-to-chronic ratios (ACR) for both fish and *Daphnia*. To address potential uncertainty parameters of the approach, the influence of the octanol–water partition coefficient Kow, the mode of action, and the acute toxicity levels on the ACR was evaluated.

**Results:**

For industrial chemicals, median ACRs of 12.0 for fish and 8.8 for *Daphnia* and 90th percentiles of 68.0 and 50.2, respectively, were determined. The ACR for the most sensitive aquatic trophic level (ACRaqu) is derived by comparing the lowest acute and chronic effect value of *Daphnia* and fish. The median ACRaqu was 9.9, and the 90th percentile was determined to 58.5. The influence of the Kow on the ACR value was analysed and a correlation could not be confirmed. Non-polar narcosis was associated with a lower ACR, whereas polar narcosis was associated with an increased ACR.

**Conclusions:**

The result suggests that an acute-to-chronic extrapolation factor of 100 is protective for more than 90 % of the chemicals. Polar narcosis may represent a predictor for an increased ACR and an increased uncertainty of the approach. The result further suggests that a high Kow is probably not associated with increased ACRs and does not necessarily represent a determinant for chronic toxicity testing within this context.

## Background

An environmental risk assessment of chemical substances (industrial chemicals, biocides, pesticides, pharmaceuticals) is based on the comparison of the predicted no-effect concentration (PNEC) and the predicted environmental concentration (PEC). The PNEC represents a concentration below which unacceptable effects are not expected and is usually derived from laboratory effect studies by applying an assessment factor on the lowest determined effect concentration or the no observed effect concentration (NOEC). According to the REACH guidance, an environmental effect assessment of the aquatic compartment requires a minimum dataset that includes results of tests with organisms from three trophic levels representing primary producers represented by algae, primary consumers, represented by invertebrates (i.e., *Daphnia*), and higher level consumers and predators, represented by fish [7, 8]. The data requirements regarding the submission of acute and chronic tests depend on the tonnage produced [5, 7].

Acute-to-chronic extrapolation is an important approach which has practical utility in current hazard assessment approaches to predict acceptable no-effect levels from acute data. The approach has its value because it saves resources and avoids animal testing. On the other hand, extrapolation from acute-to-chronic toxicity is associated with uncertainties because some chemicals may show a different mode of action (MoA) under short- and long-term conditions.

The size of an assessment factor that is used to derive a PNEC from these tests should cover several uncertainties such as intra- and interspecies variations, short- to long-term toxicity extrapolation, intra- and inter-laboratory variation or laboratory-to-field extrapolation [3, 14]. An assessment factor of 1000 is applied on the lowest effect value of acute tests from the three trophic levels of algae, invertebrates, and fish if only data from acute testing are available. This assessment factor can be refined to 100, 50, or 10 if the values of NOECs are available from long-term tests covering one, two, or three trophic levels, respectively [4, 7, 8]. Although not explicitly mentioned the procedure implies an acute-to-chronic extrapolation factor of 100 based on the difference of the assessment factor on the acute L(E)C50 value (1000) and the chronic NOEC (10) if data are available for three trophic levels.

Extrapolation from acute effect values to chronic toxicity on the basis of publicly available data has been analysed previously by several studies resulting in median acute-to-chronic ratios (ACR) ranging from 8.8 to 10.5 for fish and median ACRs ranging from 6.1 to 7.5 for invertebrates [2, 6, 9, 11–13, 22, 23]. However, conclusion on ACRs is flawed as experimental data on chronic fish toxicity are still limited with less than 40 substances being evaluated [2, 12, 23], or as most evaluations included substances such as pesticides that are expected to exert specific biological effects [6, 12, 22]. Some publications further failed to use data quality criteria and combined different species [9, 12] or included subchronic data [6]. The consideration of data points instead of substances may further influence the result by a low number of chemicals with an extensive dataset of acute and chronic studies [6].

With regard to the limited and heterogenic data of chronic fish tests, conclusions on aquatic hazard estimation need to be checked and the question still arises whether the chronic toxicity to fish can be adequately derived from acute data using fixed ACR ratios. Moreover, the ACR represents a relative value that is determined independent of the toxicity level, and that is usually not compared with the ACRs of different trophic levels for the same substance. Up-to-date, only one study compared ACRs of different trophic levels for a limited dataset of 35 substances to determine the ACR that is relevant for hazard assessment of the aquatic compartment in the EU [2].

The aim of this study was to investigate acute and chronic toxicity in *Daphnia* and fish to evaluate the acute-to-chronic extrapolation approach within the context of ecological hazard assessment. The dataset of this study is based on data gathered by [2], and extended by data of the information system chemical safety database (ICS) of the German Federal Environmental Agency (UBA) and data gathered by the OECD eChemPortal that were based on the ECHA dissemination database of REACH registrations. Data were confined to acute and chronic toxicity studies on both *Daphnia* and fish in accordance with the EU guidance documents [8] and, thus, met similar test conditions to reduce data variance. A comprehensive dataset of 203 substances including 132 industrial chemicals and 71 pesticides was gathered. The consideration of *Daphnia* and fish data for a substance allows the analysis of ACRs for the respective trophic levels as well as the evaluation of the ACR within the context of lowest acute over the lowest chronic value in line with the procedure used for aquatic hazard assessment in the EU. Data variability is further of interest to analyse possible uncertainties or potential refinement options of the extrapolation approach using a fixed ACR. Therefore, the relationship between the ACR and the MoA in terms of the acute MoA class according to the modified Verhaar scheme [24, 25], the physico-chemical property lipophilicity in terms of the octanol–water partitioning coefficient (Kow), and the acute toxicity level is investigated.

## Results and discussion

### Correlation of acute and chronic effect values

**Fig. 1:**
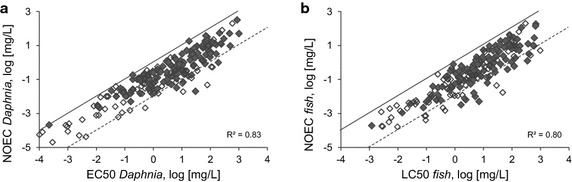
**a** Relationship between the acute (EC50) and chronic (NOEC) effect level of *Daphnia* for industrial chemicals (*filled symbol*) and pesticides (*open symbol*). **b** Relationship between the acute (LC50) and chronic effect level of fish (NOEC) for industrial chemicals (*filled symbol*) and pesticides (*open symbol*). The *upper gray line* indicates a similar acute and chronic effect value, whereas the *lower gray line* indicates a 100-fold lower chronic effect level compared to the acute effect level

Acute toxicity in terms of the EC50 value for *Daphnia* or the LC50 value for fish was correlated with the respective NOEC form the chronic study of the same substance. A relationship between acute and chronic effect values of *Daphnia* (Fig. 1a) and fish (Fig. 1b) was demonstrated for industrial chemicals. A simple regression for the dataset achieved a coefficient of determination* R*^2^ of about 0.8 for both trophic levels suggesting a good correlation between acute and chronic effect values.

### Acute-to-chronic ratios

**Fig. 2:**
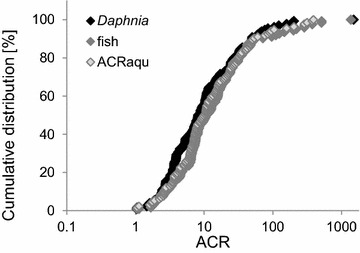
Cumulative distribution of ACRs of *Daphnia* and fish of chemicals

The cumulative distribution of ACRs is shown for *Daphnia* and fish in Fig. 2. The median ACR of fish was 12.0 with a range from 1.1 to 1371. The 90th percentile was calculated to 68.0 (Table 1). In total, eleven chemicals (9.0 %) were determined with an ACR > 100. For *Daphnia* a median ACR of 8.8 was determined with a range from 1 to 1500. The 90th percentile was 51.0 (Table 1). Six chemicals (4.6 %) had an ACR >100.

**Table 1:** Overview of results from acute-to-chronic ratio evaluation for *Daphnia* and fish

Parameter	Chemicals			Pesticides			Total		
	*Daphnia*	Fish	ACRaqu	*Daphnia*	Fish	ACRaqu	*Daphnia*	Fish	ACRaqu
Number of values	130	122	132	69	70	71	199	192	203
Minimum	1.0	1.1	1.0	1.2	1.7	1.2	1.0	1.1	1.0
10th percentile	2.4	3.5	2.8	2.3	3.0	2.3	2.5	3.1	2.7
Median	8.8	12.0	9.9	11.4	15.9	10.6	9.4	12.8	10.4
90th percentile	50.2	68.0	58.5	109.4	120.1	109.4	76.5	102.4	90.5
Maximum	1500.0	1370.6	390.0	1661.5	659.1	1661.5	1661.5	1370.6	1661.5

PNECs for the aquatic compartment are derived on the basis of the lowest effect value of at least three trophic levels [7, 8]. Therefore, the results considering the same trophic level in acute and chronic studies cannot directly be compared with the procedure used for hazard assessment in the EU. To account for this procedure an ACR that is here termed ACRaqu (ACR of the aquatic compartment) was derived by using the most sensitive trophic level in acute and the most sensitive trophic level in chronic testing of *Daphnia* and fish data. The median ACRaqu was 9.9 with a range from 1.1 to 390. The 90th percentile value was determined to be 58.5, and 6 % of the industrial chemicals had an ACRaqu >100 (Table 1). The evaluation considered *Daphnia* and fish data, although the ACR for the aquatic ecosystem has actually to consider toxicity of algae as well. Algae have been shown to often represent the most sensitive trophic level in acute testing [10], but the ACR of algae was determined with a median value of 5.4 and 90th percentile of 33.3 to be lower than that of fish or *Daphnia* [2]. Taking these findings into account, the inclusion of algae data into the dataset of this study is conceptually expected to result in comparable or lower ACR for the aquatic ecosystem. The evaluated ACRaqu based on *Daphnia* and fish data is, hence, assumed to represent a conservative measure for the ACR of the aquatic ecosystem that includes all three trophic levels.

ACRs of pesticides were separately evaluated since it is supposed that bioactive substances have a specific effect and show differences compared to industrial chemicals falling under the REACH regulation. Indeed, the evaluated ACRs of pesticides differed in their statistical profile from industrial chemicals and were increased in terms of median, 90th percentile, and maximum (Table 1).

Taken together, this study gathered a comprehensive dataset of substances with chronic effect values of fish and *Daphnia* that represent standard test species for the trophic levels invertebrates and vertebrates according to the EU guidance document. The results show a good correlation between acute and chronic effect values for both trophic levels indicating that acute-to-chronic extrapolation represents a reasonable approach for fish and *Daphnia*.

For risk assessment using acute-to-chronic extrapolation, the inclusion of a protective extrapolation factor is required and analysis of existing acute and chronic effect values is a prerequisite for the derivation of an adequate assessment factor. The evaluation of the ACRaqu represents a procedure that can be compared to the hazard assessment approach of the aquatic compartment according to the REACH guidance [7]. The determined ACRaqu as well as the ACRs of *Daphnia* and fish shows that an extrapolation factor of 100 is protective for more than 90 % of the chemicals within the scope of REACH (Table 1). Values above 1000 were only observed for ACR of *Daphnia* or fish, but not for ACRaqu. Based on this result, it is suggested that chemicals in the scope of REACH can usually be evaluated in a protective manner by the acute-to-chronic extrapolation factor of 100 as implied in the European guidance documents, although uncertainties still remain at this stage for some substance that exceed this value. Compared to chemicals, ACRs of pesticides were increased in terms of median, 90th percentile, and maximum, and exceeded the proposed assessment factor of 100 in more than 10 % of the evaluated cases. Thus, it is suggested that the extrapolation approach using an extrapolation factor of 100 is associated with an increased uncertainty for specifically acting chemicals such as pesticides.

Our results are basically comparable with previous reports, but differ in some points. Previously reported median ACRs have a range from 8.8 to 10.5 for fish and from 6.1 to 7.5 for invertebrates. Länge et al. [12] determined ACR of 6.8 for *Daphnia magna* and a median ACR of 8.8 for fish and ECETOC [6] determined a median of 6.1 for invertebrates and a median of 9.5 for fish. Both included organic chemicals, metals, and pesticides in their evaluation. Based on a LC50 to lowest observed effect concentration (LOEC) evaluation, Roex et al. [23] determined a median ACR of 6.03 for chemicals and an increased median ACR of 17.3 for specifically acting chemicals. Raimondo et al. [22] investigated the variability of species-specific ACRs including chemicals, pesticides, and metals using L(E)C50 to maximum acceptable toxicant concentration (MATC) comparison. A median ACR of 7.5 for invertebrates and a median ACR of 9.3 for fish as well as 90th percentile of 68.3 for *Daphnia* and 90.0 for fish were reported. Using an approach comparable to this study, Ahlers et al. [2] calculated a median ACR of 7.0 for invertebrates, 10.5 for fish, and 10.75 for the ecosystem based on a dataset of organic chemicals. The differences between this and previous studies are probably related to statistical data quality due to the increased number of substances with more than 200 entities on fish toxicity in the present study compared to less than 40 entities in previous studies [1, 12, 23] as well as due to the different approaches used to generate and to analyse the data (other included subchronic studies in the dataset [6], evaluated the ACR on the basis of the LOEC or MATC [22, 23], or used data point instead of substances [6], for example).

### Quality of chronic fish test types

The evaluated data of fish toxicity represented a heterogeneous dataset with a variety of species and chronic study types. Therefore, it was analysed whether differentiation between study types and same species analysis affects the result. Different quality criteria were defined and the result was analysed corresponding to the methodology applied previously [2]. The first (ACR1) included all data across all fish species and test types (Table 2). The second (ACR2) included all test types but was confined to data with acute and chronic results from the same species, whereas the third (ACR3) was confined to data from different species. ACR1, ACR2, and ACR3 showed almost comparable median values indicating that the median ACR did not differ significantly between evaluations using the same species and different species (Mann–Whitney* U* test: *p* > 0.05). However, data variance was significantly increased for interspecies ACR3 (*F* test: *p* < 0.05). ACR3 showed a 41-fold range between the 10th and the 90th percentile value compared to a 19-fold range for species-specific ACR2. This observation suggested that the variance in statistical data distribution is increased using ACR evaluations of different species for one trophic level and may affect the percentile values in statistical data analysis while the median remains almost comparable.

**Table 2:** Overview of results from acute-to-chronic ratio evaluation of chemicals for fish by different study quality criteria

Parameter	ACR1	ACR2	ACR3	ACR4	ACR5	ACR6
Number of values	122	75	47	99	23	63
Minimum	1.1	1.8	1.1	1.1	1.8	1.9
10th percentile	3.5	3.4	2.7	3.5	2.2	4.5
Median	12.0	10.8	12.8	12.7	7.3	12.2
90th percentile	68.0	63.5	111.1	96.6	36.6	63.5
Maximum	1370.6	1370.6	514.0	1370.6	375.0	1370.6

ACR1 included data across all fish species and test types

ACR2 included all test types but was confined to data with acute and chronic results from the same fish species

ACR3 was confined to data from different fish species

ACR4 was restricted to chronic toxicity testing conducted according or equivalent to OECD 210 representing FELS studies

ACR5 included all chronic studies that were not conducted according or equivalent to OECD 210

ACR6 included FELS tests that were confined to data with acute and chronic results from the same species

ACR4 was restricted to chronic toxicity testing conducted according to OECD 210, and ACR5 included non-FELS studies (juvenile growth test or the embryo and sac-fry stages test). FELS-based studies showed a median ACR4 of 12.7 and a 90th percentile of 96.6 compared to a median ACR5 of 7.3 and a 90th percentile of 36.6 for non-FELS studies. Median ACR4 of FELS tests were significantly increased compared to non-FELS ACR5 (Mann–Whitney* U* test: *p* < 0.05). Thus, the study type of the chronic test appeared to have an obvious effect on the ACR. FELS studies are considered as more conservative and appear to be more sensitive to determine chronic toxicity compared to the non-FELS studies by addressing additional endpoints and using an increased exposure period. However, the result on non-FELS studies is based on a limited dataset and interpretation should be handled with care since different test types were not compared for the same substance.

ACR6 included only FELS tests that were confined to studies with acute and chronic data from the same species. The results indicate that the acute-to-chronic extrapolation approach is applicable for the subset of data and not associated with increased uncertainties compared to the complete dataset.

### Correlation of ACR and octanol–water partitioning coefficient

**Fig. 3:**
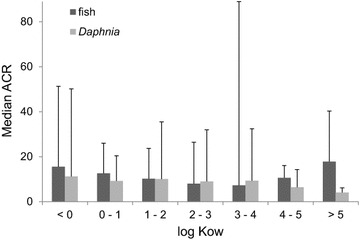
Relationship between ACR and log Kow. The median ACRs of fish and *Daphnia* were analysed for industrial chemicals using FELS test only. The indicated classes refer to one Kow magnitude. *Error bars* indicate the 75th percentile

To investigate the relationship between ACR and octanol–water partitioning, the median ACR of both *Daphnia* and fish was allocated to the indicated log Kow classes each representing one Kow magnitude (Fig. 3). A tendency for increased median values was determined for fish at a log Kow <1 and a log Kow >5. However, significant differences could not be determined for a distinct log Kow class (Mann–Whitney* U* test: *p* > 0.05 of each class compared to the remaining dataset). Based on the 75th percentile that was used instead of the 90th percentile due to the limited number of substances in each group, increased ACRs for fish were preferentially observed at a log Kow range between 3 and 4. For *Daphnia* a tendency to decreased ACRs was observed with an increase of the Kow (Fig. 3).

The evaluation of substances exhibiting a log Kow >4.5 showed that the median ACR for fish was increased compared to the complete dataset, but not significantly altered (Mann–Whitney* U* test: *p* > 0.05) (Table 3). The 90th percentile was determined to 42.6 and the ACR did not exceed 100 in any case. For substances exhibiting a log Kow >3, the result on fish as well as ACRaqu was almost comparable to the complete dataset in terms of the median and 90th percentile. For *Daphnia*, the ACR appeared to be reduced for substances exhibiting a log Kow >4.5 compared to the complete dataset (Table 3).

**Table 3:** Acute-to-chronic ratio evaluation of chemicals with a log Kow >3 and >4.5 considering data with FELS studies only

Parameter	Log Kow >3			Log Kow >4.5		
	*Daphnia*	Fish	ACRaqu	*Daphnia*	Fish	ACRaqu
Number of values	38	36	39	15	16	17
Minimum	1.1	3.2	1.1	1.5	3.4	2.2
10th percentile	2.3	5.0	2.8	2.2	7.6	3.4
Median	5.3	10.7	9.7	3.9	15.9	9.7
90th Percentile	32.9	67.0	58.5	24.6	42.6	46.4
Maximum	203	514	390	49.3	53.8	53.8

A high octanol–water partitioning coefficient is considered to be associated with the potential of bioaccumulation and a delayed steady state [4, 8]. It is assumed that the equilibrium steady state is not reached within the time frame of acute toxicity testing for substances with a high Kow. Therefore, increased toxicity is expected for substances with a high Kow upon chronic exposure resulting in increased ACRs. The evaluation of this assumption, however, has still been hampered by the limited dataset of chronic fish data. The analysed dataset comprised only FELS studies for fish so that an exposure of at least 28 days was considered in chronic testing to reach the potential steady state. The results interestingly show that median ACRs are almost comparable over the evaluated log Kow range. In line with the previous finding by Ahlers et al. [2], this result suggests that a relation between Kow and ACR cannot be established. For substances with a log Kow >4.5, the median shows a tendency and is increased by a factor of about 1.3 compared to the complete dataset (Table 3). The change is, however, associated with an increase of the 10th percentile from 3.5 to 7.6. On the other hand, the 90th percentile is reduced compared to the complete dataset suggesting a reduced uncertainty at increased log Kow values. Thus, it can be concluded that increased ACRs are probably not associated with high Kow values and chronic testing is not more compelling for substances with a log Kow >3 or >4.5 than for other substances within this context. This finding is controversial to the current assumption and requires further investigation due to the limited dataset of substances with a log Kow >4.5. The observation may be explained by mechanistic reasoning since it can be supposed that largely increased ACRs (e.g., ACR >100) are probably independent of the Kow and may result from the evaluation of different MoA due to the evaluation of life stages and additional endpoints in chronic tests that are not covered by acute tests.

### Relationship between acute toxicity and ACR

**Fig. 4:**
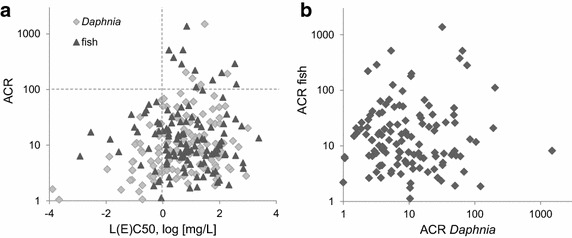
**a** The ACR of fish and *Daphnia* of the complete dataset of chemicals was correlated with the respective acute effect concentration (EC50, LC50). **b** The ACR of fish and *Daphnia* was correlated for each substance

ACRs of *Daphnia* and fish were correlated with the acute L(E)C50 toxicity level to investigate the relationships between ACR and compounds with highly acute toxicity (Fig. 4a). 90th percentiles of 60.5 and 111.1 were determined for *Daphnia* and fish for substances exhibiting L(E)C50 values of >1 mg/L, whereas reduced values were determined for substances exhibiting acute L(E)C50 values <1 mg/L. ACR values above 100 were only observed for substances exhibiting acute values of L(E)C50 >1 mg/L (Fig. 4a).

Previously, it was hypothesized that highly acute toxic compounds have little room to increase through prolonged exposure times [2]. Moreover, it can be supposed that some chemicals showing less acute toxicity may address non-lethal endpoints covered by chronic tests. The results of this study indicate that highly acute toxic compounds with L(E)C50 <1 mg/L have a lower probability for substantially increased ACR values. This suggests that the uncertainty of the extrapolation approach is expected to be reduced for substances with an L(E)C50 <1 mg/L. Nevertheless, several substances exhibiting acute L(E)C50 values <1 mg/L exhibit an ACR value between 10 and 100.

### Correlation of ACRs of fish and *Daphnia*

ACRs of *Daphnia* and fish were further correlated to investigate whether predictions of the ACR between trophic levels are applicable to refine the hazard assessment if data of one trophic level are not available (Fig. 4b). A correlation between ACRs of *Daphnia* and fish could not be established and ACRs were significantly different for *Daphnia* and fish (Wilcoxon test: *p* < 0.05). This finding suggests that it is generally not possible to conclude from *a Daphnia* ACR on fish ACR, and vice versa.

### Mode of action (MoA) classification

To analyse the relationship between acute MoA and the ACR, classification according to the modified Verhaar scheme was applied that is designed to predict acute MoA of organic chemical in fish [25]. Non-polar narcotics (MoA1) represent a group of chemicals that are considered as inert with regard to chemical or biological reactivity. MoA2 comprise polar non-reactive substances such as anilines, phenols, or substances that are characterized by possessing hydrogen bonds and may ionize to some extent depending on the pH. Reactive substances (MoA3) represent a diverse group of chemicals with different unspecific reaction mechanisms that are assumed to result in enhanced toxicity compared to baseline toxicity. Substances that are expected to exert a specific mechanism (MoA4) were not determined in the group of industrial chemical confirming the initial subdivision of the dataset into chemicals and pesticides. Chemicals within the applicability domain which could not be assigned to one of the MoA classes were grouped in MoA5 according to the algorithm of the OECD toolbox.

The range of median ACRs was determined between 7.5 and 51.9 and the range of 90th percentile was between 24.1 and 514.0 for the different MoA classes (Table 4). Compared to other MoA classes and compared to the result for the complete dataset, substances assigned to MoA2 exhibited increased ACRs for fish. The median ACR and 90th percentile clearly exceeded a value of 10 and 100, respectively. MoA1 showed decreased ACR values. MoA3 and MoA5 exhibited a slightly reduced median and 90th percentile compared to the complete dataset, and the maximum did not exceed a value of 100.

**Table 4:** Relationship between MoA and ACR

	MoA1	MoA2	MoA3	MoA5	Total
Number of values	23	18	12	30	83
Minimum	2.2	4.5	2.7	1.9	1.9
Median	7.6	51.9	9.6	10.8	12.0
90th percentile	24.1	514.0	56.0	39.1	67.0
Maximum	40.1	1370.6	63.5	67.0	1370.6

The finding that the ACR for polar narcotics (MoA2) appeared to be increased, whereas the ACR for non-polar narcotics (MoA1) was determined to be reduced compared to the complete dataset is in line with Raimundo et al. [22]. This result suggests that non-polar narcosis represents a predictor for lower ACR, whereas polar narcosis represents a predictor for increased ACR.

## Conclusions

Acute-to-chronic extrapolation is an important approach to predict acceptable no-effect levels from acute data which has some uncertainties, but is valuable for risk assessment of chemical substances. The present study comprises a comprehensive dataset of studies on acute and chronic toxicity of *Daphnia* and fish that have been conducted in line with OECD guidelines recommended in the EU guidance documents. The evaluation of existing data on *Daphnia* and fish toxicity testing shows that acute-to-chronic extrapolation represents a reasonable approach for environmental hazard estimation by considering an adequate assessment factor. Based on the evaluated dataset, an AF of 100, as implied by the European guidance documents, is protective for more than 90 % of industrial chemicals to estimate *Daphnia* and fish toxicity. Moreover, a value of 1000 applied on the lowest acute effect level of a substance was not exceeded in any case for the aquatic compartment (ACRaqu). Data variability is of interest to predict uncertainties and refinement options. The study indicates that bioactive compounds such as pesticides as well as polar narcotics show increased ACRs. On the other hand, non-polar narcosis is associated with reduced ACRs. The physico-chemical parameter Kow is of minor value to predict increased ACRs or to address uncertainties of this approach, while the acute toxicity level may provide options for refinement of acute-to-chronic extrapolation.

## Methods

Data were gathered by using the OECD eChemPortal, and the information system chemical safety database (ICS) of the German Federal Environmental Agency (UBA). The dataset generated by the OECD eChemPortal was based on the ECHA dissemination database. The entries of this database were generated for the chemical registration in the EU under the REACH regulation. Data from the ECHA database are provided by the registrants. Thus, the primary data source could often not be evaluated. The data are assigned to the Klimisch quality score and data with Klimisch 1 or 2 were selected. Data gathered in a previous project by Ahlers et al. [2] as well as data provided by the ICS database comprising a dataset of substances notified under the “New Chemicals” legislation (67/548/EEC) were validated by the German UBA. The data provided by industry are confidential or possess protection claims so it is not possible to publish the data in detail.

The dataset is confined to standardized tests and includes acute and chronic data on both fish and *Daphnia* for each substance entry to allow the evaluation of ecotoxicological risk estimation within the context of the European Union chemical registration. Selection of data is based on studies that were conducted according to the recommended OECD guidelines. Selection criteria were conformity in species, endpoints investigated, test system, and test duration. If more than one study was documented for an endpoint, the lowest effect concentration was considered as this is usually chosen as key study and relevant for risk assessment. Effect values above the water-solubility limit as well as open-ended toxicity values were not included in the data analysis.

Short-term fish toxicity was ascertained from the 96-h median lethal concentration (LC50) of tests performed according to OECD 203 or comparable design [16]. For long-term tests on fish, the fish early-life stage test (FELS) according or equivalent to OECD 210 or life-cycle tests was used [21] considering the no-observed effect concentration (NOEC) with the endpoints hatching and survival, length, weight or abnormal behaviour as recommended in the OECD 210 guideline. In addition, the embryo and sac-fry stages according to OECD 212 (for substances with a log Kow <4) as well as the fish juvenile growth test according to OECD 215 (for substances with a log Kow <5) were used to evaluate chronic toxicity [18, 19]. Non-guideline studies were considered only if a documented study met the criteria for guideline requirements of the respective guideline. Read across, in general, and studies on adult fish such as testing according to OECD 204 were not considered [15]. Fish studies were preferentially selected based on the recommended species *Pimephales promelas, Danio rerio, Oncorhynchus mykiss*, and *Oryzias latipes* as given in the OECD 210 guidance document [21]. Data for which both acute and chronic results were available for the same species were preferentially considered. To further consider the broad variety of available data on fish, ACR on different fish species was also included. For comparative purpose of the test type and to distinguish between same or different fish species evaluation, a separate analysis was performed with specified subgroups.

For acute tests on invertebrates, studies conducted according or equivalent to the OECD Guideline 202 (*Daphnia* acute test) using the 48 h median effective concentration (EC50) value were considered [20]. Chronic invertebrate tests according or equivalent to OECD 211 (*Daphnia* chronic test) were considered using the 21 days NOEC [17]. Thereby, the most sensitive effect value from the endpoints reproduction or mortality (immobilization) was used. Studies on invertebrates were primarily conducted on *D. magna* and in single cases on *D. pulex*. Other invertebrate species were excluded to reduce data variance.

The dataset for ACR evaluation included 203 substance entities that were considered within the scope of this study. The initial dataset comprised active substances that are applied as pesticides and expected to exert specific biological effects on organisms. Pesticides and chemicals in the scope of REACH were subdivided to account for differences between both with regard to different regulatory requirements and to address potential differences with regard to the results of this study. Subsequently, active substances that have been notified as plant protection products or pesticides were separated from the dataset resulting in 70 pesticides and 132 industrial chemicals that were registered under the REACH regulation or the “New Chemicals” legislation (67/548/EEC).

The ACR was calculated as the ratio of the L(E)C50 from acute tests on *Daphnia* or fish to the NOEC from the most sensitive endpoint of the respective chronic study. The resulting ACR distribution deviated from normal distribution and was analysed by non-parametric tests using Statistica (StatSoft, OK, USA).

Model-derived MoA classification of the dataset into the classes according to Verhaar [25] was obtained using an implementation in the OECD QSAR Toolbox 3.2 (http://www.oecd.org/chemicalsafety/risk-assessment/theoecdqsartoolbox.htm).
